# New solvation free energy function comprising intermolecular solvation and intramolecular self-solvation terms

**DOI:** 10.1186/1758-2946-5-8

**Published:** 2013-02-04

**Authors:** Hwanho Choi, Hongsuk Kang, Hwangseo Park

**Affiliations:** 1 Department of Bioscience and Biotechnology, Sejong University, 98 Kunja-dong, Kwangjin-ku, Seoul, 143-747, Korea

**Keywords:** Solvation free energy, Self-solvation, Solvent-contact model, Genetic algorithm, Atomic parameters

## Abstract

Solvation free energy is a fundamental thermodynamic quantity that should be determined to estimate various physicochemical properties of a molecule and the desolvation cost for its binding to macromolecular receptors. Here, we propose a new solvation free energy function through the improvement of the solvent-contact model, and test its applicability in estimating the solvation free energies of organic molecules with varying sizes and shapes. This new solvation free energy function is constructed by combining the existing solute-solvent interaction term with the self-solvation term that reflects the effects of intramolecular interactions on solvation. Four kinds of atomic parameters should be determined in this solvation model: atomic fragmental volume, maximum atomic occupancy, atomic solvation, and atomic self-solvation parameters. All of these parameters for total 37 atom types are optimized by the operation of a standard genetic algorithm in such a way to minimize the difference between the experimental solvation free energies and those calculated by the solvation free energy function for 362 organic molecules. The solvation free energies estimated from the new solvation model compare well with the experimental results with the associated squared correlation coefficients of 0.88 and 0.85 for training and test sets, respectively. The present solvation model is thus expected to be useful for estimating the solvation free energies of organic molecules.

## Background

Solvation free energy serves as a characteristic property of various molecules in material, biological, and pharmaceutical sciences. For example, the knowledge of solvation free energy is prerequisite for the determination of the equilibrium constant for protein-ligand association because the desolvation costs for complexation can make a significant contribution to the total binding free energy [1]. Solvation properties are also important in drug discovery because they have an effect on the bioactivity of drug candidates at the site of action. This renders the determination of molecular solvation free energy or solubility necessary at the early stage of drug discovery [2]. However, the experimental measurement of solvation free energy is a time-consuming procedure, which makes it very difficult to screen a large chemical library from which physically or biologically active compounds can be identified. The need to estimate the differences between solvation free energies of structurally related compounds has become more urgent in recent years with the advent of combinatorial chemistry [3], further necessitating the development of a reliable computational method to predict solvation free energies of organic molecules.

However, the solvation free energy has been considered one of the most calculation-difficult energy terms due to the complexity of solute-solvent interactions [4]. Many computational methods for the prediction of molecular solvation free energies have nonetheless been explored since the earlier work of Onsager [5]. The simplest solvation model could be represented by the adjustment of the dielectric constant in a distant-dependent way to mimic the electrostatic screening by solvent [6]. More precise methods for predicting molecular solvation free energies than the dielectric continuum models were also suggested on the basis of the Poisson-Boltzmann equation to calculate the electrostatic potentials around the solute molecule [7]. These implicit solvation models were actually incapable of reflecting the solute-solvent interactions on atomic scale, which has an effect of limiting the reliabilities of the calculated molecular solvation free energies. On the other hand, the all-atom model calculations based on molecular dynamics and Mote Carlo simulations have proved to be useful for the precise estimation of molecular solvation free energies [8-12], even in the case of protein-ligand complexes [13]. Molecular solvation free energies have also been estimated with accuracy from various high-level quantum chemical calculations [14-17]. Despite the improved accuracy, however, the high computational costs have prevented the quantum mechanical and the all-atom models for molecular solvation from being employed widely in practical applications [18,19]. As a compromise between the computational cost and the accuracy, a variety of efficient computational methods with reasonable accuracy have been proposed based on various theoretical frameworks such as solvent-accessible surface area model [20,21], 3-D reference interaction site model [22], cellular automata based algorithm [23], quantitative structure–property relationship (QSPR) model [24], linear interaction energy method [25], and quantum mechanical continuum solvation models [26].

In the early 1990s, Stouten et al. proposed a solvation free energy function on the basis of the solvent-contact model developed by Colonna-Cesari and Sander [27,28]. Under the assumption that molecular solvation free energy could be given by the sum over the individual atomic contributions, they optimized the atomic parameters in the solvation energy function for the six atom types (C, N, O, N^+^, O^-^, and S). Although this simple solvation model proved to be successful in estimating the structural properties of proteins in solution as well as in saving the computational time [28], its applicability could not be extended to organic molecules because the number of atom types was insufficient to discriminate the atoms with a variety of chemical environments. Therefore, we improved Stouten et al.’s solvation model in the previous study by extending the atom types to cope with various small organic molecules [29]. The modified solvation free energy function defined with 69 atomic parameters for 23 atom types was shown to estimate the solvation free energies of small organic molecules with reasonable accuracy.

In this study, we propose a new solvation free energy function by further improving the solvent-contact model in terms of the two points. First, the previous solvation models were developed on the basis of the group additivity that assumed a linear relationship between the solvation free energy and the volume of hydration shell. However, this assumption was shown to be inappropriate when the solvation free energy could be affected significantly by the intramolecular interactions between solute atoms [30,31]. This is called the self-solvation and was found to be an important factor that should be considered in modeling proteins in solution [32]. By examining the self-solvation effects on molecular solvation free energies, we aim to obtain a new solvation free energy function that can reflect the nonadditivity inherent in solute-solvent interactions. Second, the space of atom types needs to be extended to differentiate the atoms in organic molecules with varying molecular sizes and shapes. The solvation free energy function was indeed shown to become more accurate by the subdivision of the atom types in such a way to fully describe the complex chemical environments [29]. Furthermore, most of the existing solvation models have been developed and optimized with small organic molecules only due to the lack of the experimental solvation energy data for large molecules. This limited their applicability to the molecules with low molecular weight. Prior to the development of a new solvation free energy function, therefore, we constructed a new dataset for solvation free energy of organic molecules with molecular weights ranging from 200 to 500 amu using their experimental data for aqueous solubility and vapor pressure. Thus, we aim to establish a new solvation free energy function involving the self-solvation effects and extended atomic parameters using the molecules with varying sizes and shapes.

## Methods

### Construction of the new solvation free energy function

The solvent-contact model for molecular solvation is based on several assumptions. First, the solvation free energy (*ΔG*_*sol*_) of a molecule can be approximated by the sum of individual atomic contributions as follows.

(1) $$\Delta G_{\mathrm{sol}}=\sum_{i}^{\mathrm{atoms}}\Delta G_{\mathrm{sol}}^{i}$$

Second, the solvation free energy of an atom *i* can be given by the product of the atomic solvation parameter (*S*_*i*_) and its volume exposed to bulk solvent (*F*_*i*_).

(2) $$\Delta G_{\mathrm{sol}}^{i}=S_{i}F_{i}$$

Third, the atomic volume exposed to solvent is assumed to be equal to the unoccupied volume around the atom of interest. The occupied volume around the atom *i* (*O*_*i*_) indicates the region to which the approach of solvent molecule is forbidden due to the occupation by the other solute atoms. *O*_*i*_ can be determined by summing over the product of an atomic volume parameter (*V*_*j*_) representing the fragmental volume of the other atoms and a suitable envelope function, *E*(*r*_*ij*_), with respect to the distance between the centers of atoms *i* and *j*.

(3) $$O_{i}=\sum_{j\neq i}^{\mathrm{atoms}}V_{j}E\left(r_{\mathrm{ij}}\right)$$

Here, Gaussian envelope function [28] is used with the variable *r*_*ij*_ representing the interatomic distance and the σ value of 3.5 Å. Because *F*_*i*_ is the difference between the maximum occupancy of atom *i* (*O*_*i*_^*max*^) and *O*_*i*_, the solvation free energy of a molecule can be expressed in the following form.

(4) $$\Delta G_{\mathrm{sol}}=\sum_{i}^{\mathrm{atoms}}S_{i}\left(O_{i}^{\max}-\sum_{j\neq i}^{\mathrm{atoms}}V_{j}e^{-\frac{r_{\mathrm{ij}}^{2}}{2\sigma^{2}}}\right)$$

Although the solvation free energies of small organic molecules calculated with Equation (4) compared well with experimental results, this solvation model needs to be modified in order to be useful in practical applications because of the neglect of the self-solvation effect. Indeed, it has been demonstrated that the effects of the intramolecular non-bond interactions between solute groups should be reflected in the solvation free energy function to describe the solute-solvent interactions in a quantitative fashion [30-33]. For example, the intramolecular hydrogen bond and van der Waals interactions established in the occupied volume of the solute can affect the strength of the solute-solvent interactions through the change in electron distribution in the outer region exposed to bulk solvent. The presence of this self-solvation effect can be attributed to the intramolecular stabilization/destabilization of the atoms in the solvent-exposed region by the atoms in the occupied volume. The pattern for solute-solvent interactions can thus be affected significantly by the intramolecular interactions that may lead to the charge redistribution at the solute-solvent interface.

**Figure 1:**
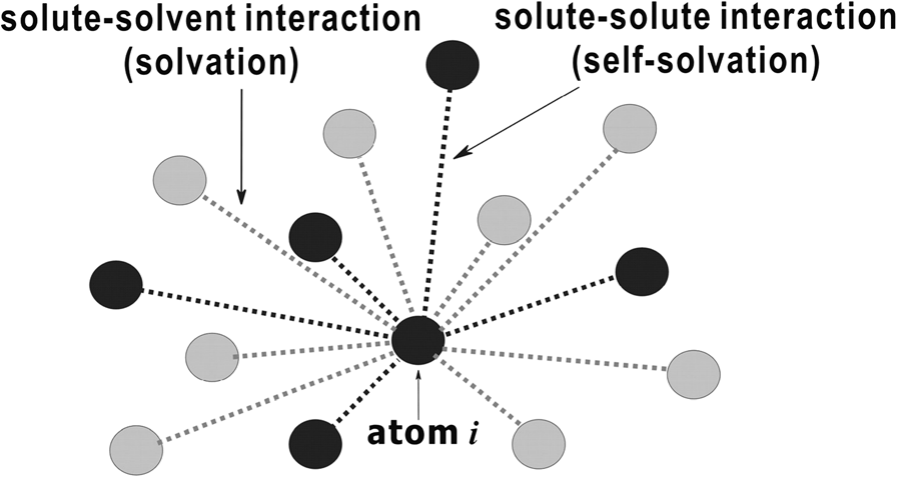
**Schematic diagram for the interactions of solute atom *i *in solution.** The black and gray circles indicate solute and solvent atoms, respectively. In this example, the atom *i* interacts with eight solvent atoms and five the other solute atoms.

Figure 1 describes a typical pattern for the interactions of solute atoms in solution. As illustrated, a solute atom can be stabilized not only by the interactions with solvent molecules (solvation) but also by those with the rest of solute atoms (self-solvation). Therefore, the solvation energy function in Equation (4) should be insufficient to fully describe the stabilization of a solute molecule in solution because it contains the solute-solvent interaction term only and lacks the self-solvation term.

To define the self-solvation energy of an organic molecule in solution, we assume that it can be obtained by the summation of all individual atomic contributions. The self-solvation energy of an atom *i* (*ΔG*_*self*_^*i*^) can then be approximated by the product of the atomic self-solvation parameter (*P*_*i*_) and the occupied volume around the atom *i* as follows.

(5) $$\Delta G_{\mathrm{self}}^{i}=P_{i}O_{i}$$

Here, *P*_*i*_ describes the stabilization energy of solute atom *i* per unit volume due to the intramolecular interactions with the other atoms of the solute. The self-solvation term can be appended to the solvation term in Equation (4) to obtain the improved solvation free energy function, which can be expressed in the following form.

(6) $$\Delta G_{\mathrm{sol}}=\sum_{i}^{\mathrm{atoms}}\left\{S_{i}\left(O_{i}^{\max}-\sum_{j\neq i}^{\mathrm{atoms}}V_{j}e^{-\frac{r_{\mathrm{ij}}^{2}}{2\sigma^{2}}}\right)+P_{i}\sum_{j\neq i}^{\mathrm{atoms}}V_{j}e^{-\frac{r_{\mathrm{ij}}^{2}}{2\sigma^{2}}}\right\}$$

The first and second terms in Equation (6) correspond to the contribution from solute-solvent interactions and that from the intramolecular interactions between solute atoms to the stabilization of the solute molecule in solution, respectively. This new solvation free energy function places an emphasis on the fact that an organic solute molecule can be stabilized in solution as a consequence of the coordination between the solute-solvent interactions and the stabilization of its internal structure. The four key atomic parameters in the present solvation model include the maximum atomic occupancy (*O*_*i*_^*max*^), the atomic fragmental volume (*V*_*i*_), and the atomic solvation (*S*_*i*_) and self-solvation (*P*_*i*_) energies per unit volume. The extent of contribution from the intramolecular interactions to molecular solvation free energy can thus be determined by *P*_*i*_ parameters in the present solvation model. The negative and positive values of *P*_*i*_ parameter indicate the stabilization and destabilization of the solute atom *i*, respectively, due to the intramolecular interactions with the rest of solute atoms. Thus, four different atomic parameters should be optimized for all possible atom types to obtain a complete form of the solvation free energy function. It should be noted that molecular solvation free energies can be calculated in a straightforward way using the 3-D molecular structures and the optimized atomic parameters only. Therefore, the computational cost in the present solvation model can be saved to a significant extent when compared to the other methods that require the quantum chemical calculations or statistical modeling. Due to the reduction in computing time, the present solvation model can be an appropriate tool for coping with large chemical libraries.

### Data set

To complete the solvation free energy function, it was required to prepare a reference dataset with which the atomic parameters could be optimized. Therefore, we constructed a chemical library containing 404 organic molecules from Physical/Chemical Property Database (PHYSPROP) [34] and Hazardous Substances Data Bank [35] in which experimental vapor pressure and solubility data were available. Using these experimental data, solvation free energies in dilute solution were obtained from the following relation [36].

(7) $$\Delta G_{\mathrm{sol}}=-2.303\mathrm{RT}\log\left[\frac{M}{p/p^{0}}\right]$$

Here, *M* and *p* represent the equilibrium solubility and vapor pressure of a molecule measured at 298.15 K and 1 atm for a pure solid solute, respectively, while *p*^*0*^ denotes the pressure of ideal gas at 1 M and 298.15 K. To validate the accuracy of Equation (7), we examined the similarity of the estimated solvation free energies to the experimental ones using 199 molecules for which experimental data of solvation free energy, *M*, and *p* were available [37]. The linear correlation coefficient between the experimental and estimated molecular solvation free energies amounts to 0.97 with the associated slope and intercept values of 1.04 and 0.13, respectively. This high correlation indicates that the molecular solvation free energies obtained with Equation (7) may be sufficient to serve as a dataset for parameterization.

Total 404 molecules were then divided into 362 and 42 elements at random to construct the training and test sets, respectively. To obtain their 3-D atomic coordinates, we used the CORINA program [38] with which a stable conformation of each molecule was generated on the basis of the conformational parameters derived from the X-ray crystal structures of small molecules. The prepared 3-D structures of the molecules were refined with quantum chemical geometry optimizations at B3LYP/6-31G* level of theory to obtain the final structures from which molecular solvation free energies were calculated. For simplicity, only single molecular conformation was considered in this study although multiple conformations for a molecule should be taken into account to further improve the solvation free energy function.

### Definition of atom types

Because different atom types make different contributions to solvation free energy, the atom types in a molecule should be differentiated according to the element, hybridization state, and chemical environment around the atom under consideration. Previously we defined 23 basic atom types for the atoms commonly found in small organic molecules based on the element and the hybridization state. In the present solvation model, we extend the set of atom types to include 37 elements by subdividing the atom types according to the number of substitutions as well as to the element and the hybridization state. This subdivision of the atom types seemed to result in the improvement of the solvation free energy function due to the reflection of varying solvent accessibilities around a solute atom. Considering the portability and the simplicity for implementing the atom type classifications, all atom types were designated in the same fashion as in the Sybyl MOL2 format.

### Optimization of atomic volume parameters with genetic algorithm

**Figure 2:**
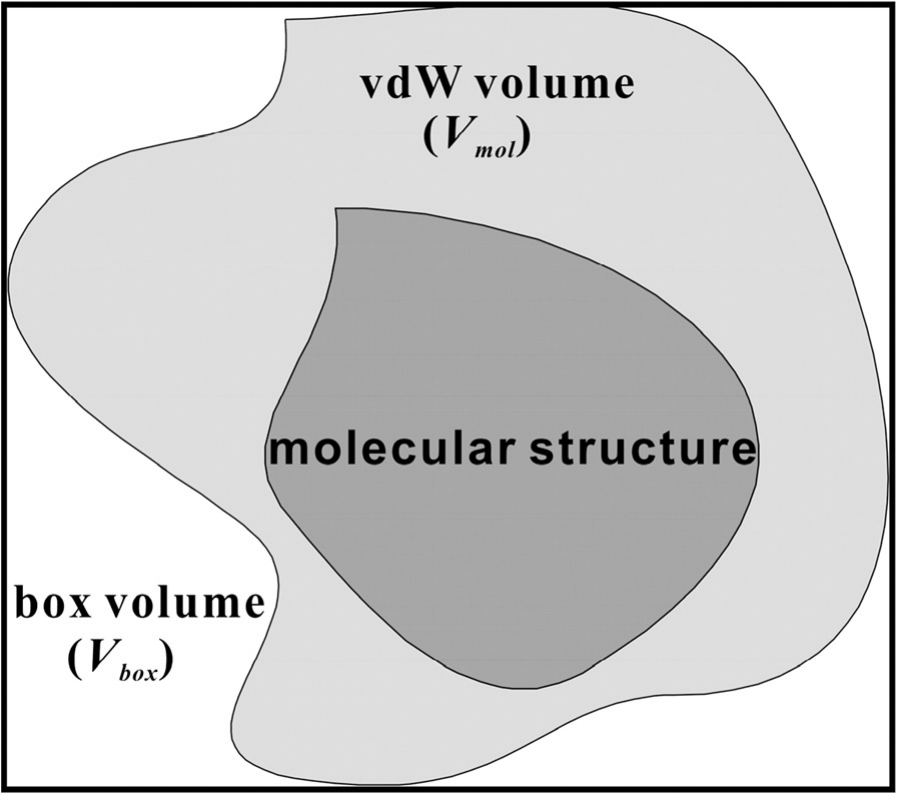
**Embedment of a molecule in a box to calculate the total volume (*V_mol_*).**

As mentioned above, four atomic parameters need to be determined for all atom types to obtain a complete form of the solvation free energy function. Among them, the atomic volume parameter *V*_*j*_ represents the fragmental volume of atom *j* in a molecule. Because these *V*_*j*_ values exhibited a bad convergent behavior in the simultaneous optimization of the four kinds of parameters, they were optimized separately with the genetic algorithm as detailed below. The determination of molecular volumes (*V*_*mol*_’s) of individual molecules was required to optimize *V*_*j*_ parameters for varying atom types. To calculate the *V*_*mol*_ values, each molecule was placed in a 3-D box as illustrated in Figure 2. The length, width, and height of this box correspond to the maximum distances along the three axes defining the coordinate system of the van der Waals volume of the molecule. To define the molecular van der Waals volumes, atomic radii of carbon, nitrogen, oxygen, sulfur, hydrogen, fluorine, chlorine, bromine, and iodine atoms are set equal to 1.53, 1.45, 1.36, 1.70, 1.08, 1.30, 1.65, 1.80, and 2.05, respectively. Monte Carlo simulations involving the random selections of a point in the predefined 3-D box were then carried out to calculate the *V*_*mol*_ value of the molecule embedded in the box. In this simulation, *V*_*mol*_ could be obtained by the product of the box volume (*V*_*box*_) and the ratio of the number of trials to select a point in the van der Waals volume (*N*_*hits*_) to the total number of trials (*N*_*trials*_). All *V*_*mol*_ values for the molecules in the dataset were thus obtained using the following equation.

(8) $$V_{\mathrm{mol}}=V_{\mathrm{box}}\times \frac{N_{\mathrm{hits}}}{N_{\mathrm{trials}}}$$

Although the *V*_*j*_ parameters of a single molecule can also be determined by the Monte Carlo simulations described above, they may be varied with the change of molecules with different sizes and shapes. To obtain the *V*_*j*_ values that represent the average atomic contributions with the atom type *j* to the van der Waals volumes of various molecules, therefore, they were optimized with the standard genetic algorithm using the calculated *V*_*mol*_ values of all molecules under consideration. This started with the definition of a generation defined with 100 vectors comprising the *V*_*j*_ parameters for all atom types, which was followed by the removal of 50 with a bias toward preserving the most fit with the lowest error. The empty 50 vectors were then filled with point mutations to alter the value of one of the parameters with probability 0.01, and with cross breeds with probability 0.6 to select some parameters from one vector to replace the elements of another vector of the top 50. The 50 new vector created in these ways were then evaluated together with the top 50. This cycle was repeated as many times as desired. To evaluate the 100 vectors, we used the error hypersurface (Fv) defined by the sum of the absolute values of the differences between the calculated Vmol value and the sum of Vj values of a molecule.

(9) $$F_{V}=\sum_{k}^{\mathrm{molecules}}\left|V_{\mathrm{mol}}^{k}-\sum_{j}^{\mathrm{atoms}}V_{j}\right|$$

### Optimization of atomic solvation and self-solvation parameters

In addition to *V*_*j*_, three remaining atomic parameters (*S*_*i*_, *O*_*i*_^*max*^, and *P*_*i*_) in Equation (6) should be determined for each atom type to obtain the complete form of solvation free energy function. These parameterizations were carried out by operating the genetic algorithm with the same procedure as in the optimization of *V*_*j*_ parameters. To optimize the parameters, the error hypersurface was defined by the sum of the absolute values of the differences between the molecular solvation free energy measured from experiment (*ΔG*_exp_^*i*^) and that estimated with Equation (6) (*ΔG*_*calc*_^*i*^). This fitness function can be written as follows.

(10) $$F_{s}=\sum_{i=1}^{\mathrm{molecules}}\left|\Delta G_{\exp}^{i}-\Delta G_{\mathrm{calc}}^{i}\right|$$

During the operation of genetic algorithm, the atomic parameters exhibited convergent behavior after 10,000 iterations with the *F*_*s*_ value of 0.73 kcal/mol.

## Results and discussion

Prior to the calculation of solvation free energies of 404 organic molecules, their geometries were fully optimized at B3LYP/6-31G* level of theory from the initial structures generated with the CORINA program. With the final 3-D structures of 404 molecules and their experimental solvation free energy data in hand, we first evaluated the previous solvation model that neglected the self-solvation effect and considered 23 atom types only. Listed in Table 1 are the atomic volume (*V*_*j*_), maximum atomic occupancy (*O*_*i*_^*max*^), and atomic solvation parameters (*S*_*i*_) in Equation (4) for 23 atom types that were optimized with 362 molecules in the training set. In contrast to the *O*_*i*_^*max*^ values, the optimized *V*_*j*_ values exhibit a large difference with varying atom types even in the case of the same element. For example, the *V*_*j*_ values of sp^3^ and sp^2^ sulfur atoms appear to be even larger than those of sulfoxide and sulfone groups in contrast to the similarity in *O*_*i*_^*max*^ values for all sulfur atoms. Actually, such a large difference in *V*_*j*_ parameters of the similar atoms is not surprising because each *V*_*j*_ value represents the average of atomic contributions with type *j* to the van der Waals volumes of the molecules with various sizes and shapes.

**Table 1:** **The optimized atomic fragmental volume (*V_j_*), maximum atomic occupancy (*O_i_***^**max**^**), and atomic solvation parameters (*S_i_*) in the solvation model without self-solvation effects**

**Atom type**	**Description**	***V***_***j***_**(Å**^**3**^**)**	**O**_***i***_^**max**^**(Å**^**3**^**)**	***S***_***i***_**(kcal/molÅ**^**3**^**)**
C.3	sp^3^ carbon	8.276	322.8	1.619
C.2	sp^2^ carbon	8.571	328.9	−0.730
C.1	sp carbon	10.952	335.5	−1.958
C.ar	aromatic carbon	8.968	352.4	−0.036
N.3	sp^3^ nitrogen	6.984	326.4	−0.938
N.2	sp^2^ nitrogen	8.344	328.7	−3.952
N.1	sp nitrogen	8.622	364.3	−4.857
N.am	amidic nitrogen	8.462	357.8	−8.439
N.ar	aromatic nitrogen	8.133	338.3	−2.707
N.pl3	trigonal planar nitrogen	8.175	331.8	−6.063
O.3	sp^3^ oxygen	6.851	368.5	−5.429
O.2	sp^2^ oxygen	7.381	344.3	−4.968
S.3	sp^3^ sulfur	16.856	340.6	−0.905
S.2	sp^2^ sulfur	17.619	348.3	2.254
S.O	sulfoxide sulfur	13.547	345.2	−2.159
S.O2	sulfone sulfur	13.563	338.2	−0.952
P	phosphorine	12.381	330.3	−1.841
F	Fluorine	6.190	327.0	−2.143
Cl	Chlorine	16.325	327.3	−0.397
Br	Bromine	22.064	330.4	0.714
H.C	hydrogen bonded to carbon	3.143	367.3	0.905
H.N	hydrogen bonded to nitrogen	2.571	364.8	−4.381
H.O	hydrogen bonded to oxygen	2.763	362.1	−7.429

The optimized *S*_*i*_ parameters exhibit a trend consistent with general atomic properties. We note, for example, that the *S*_*i*_ values become more negative in going from sp^3^ to sp^2^ and sp in cases of carbon and nitrogen atoms. This indicates that the atomic solvation should become more favorable with the increase of the s-character in the hybridization state of the solute atom. Such a dependence of *S*_*i*_ value on the degree of s-character can be understood because the increase in the s-character of the hybrid orbitals of a central atom leads to the increase in its electronegativity and culminates in the promotion of dipole-dipole interactions with solvent molecules. The amidic nitrogens appear to have the most negative *S*_*i*_ value. This is consistent with the delocalization of its lone-pair electrons to the neighboring aminocarbonyl oxygen, which has an effect of increasing the polarity of the amide group. In case of hydrogen atoms, *S*_*i*_ values are found to become more negative in the order of the electronegativity of the heavy atom to which the hydrogen of interest is attached. This can also be understood by noting the fact that the increase in the electronegativity causes to enhance the acidity of the central atom, which would have an effect of strengthening the hydrogen-bond interactions with solvent molecules. It is thus apparent that in the absence of self-solvation effects, the electronegativities of the solute atoms can serve as a key factor for the sign and magnitude of *S*_*i*_ parameters.

**Figure 3:**
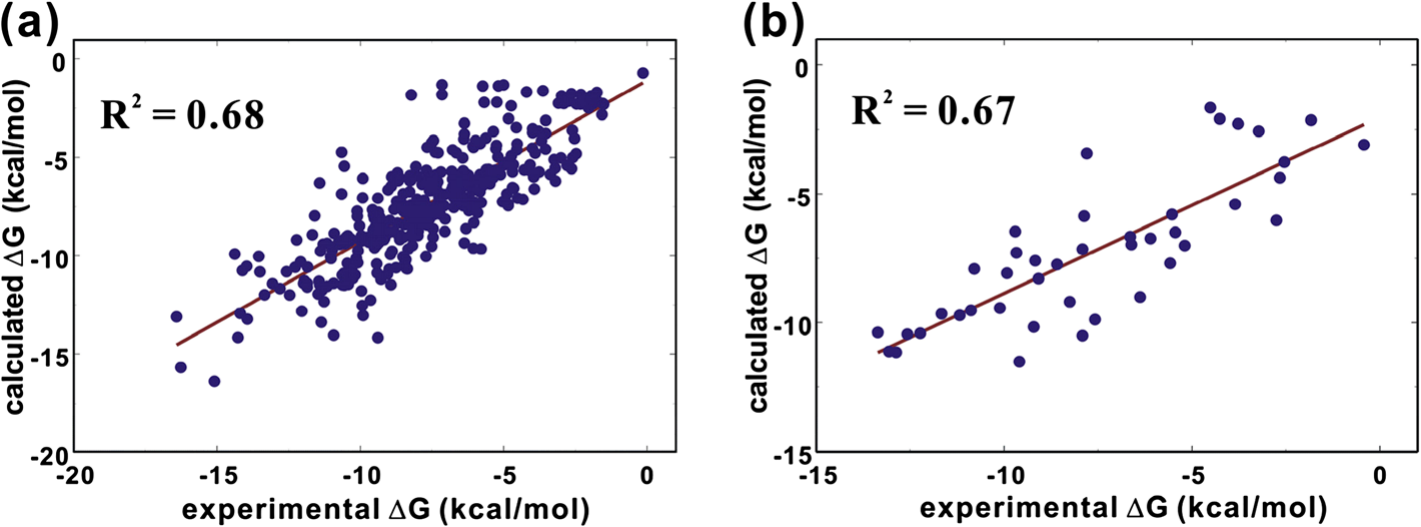
**Correlation diagrams for the experimental solvation free energies (ΔG) versus those obtained with the solvation free energy function without self-solvation term for (a) 362 molecules in the training set and (b) 42 molecules in the test set.** The slope and intercept of the fitting for the test set are 0.98 and −0.52, respectively.

The correlations between the solvation free energies measured from experiments and those obtained with Equation (4) are illustrated in Figure 3. With the test set comprising 42 molecules, we obtain the squared correlation coefficient (*R*^*2*^) of 0.67, which is similar to that of the fitting with the training set including 362 molecules (0.68). In the case without reoptimizing the atomic parameters, the *R*^*2*^ value for the test set falls to 0.58. The calculated solvation free energies of the compounds under consideration are thus found to be inaccurate as compared to those for small organic molecules with molecular weights ranging from 30 to 180 amu for which *R*^*2*^ values amount to 0.89 and 0.86 for training and test sets, respectively [27]. The reason for such a significant decrease in the accuracy lies in that the dataset employed in this study comprises relatively large organic molecules with molecular weights ranging from 200 to 500 amu. The previous solvation model described in Equation (4) is thus shown to be useful only for estimating the solvation free energies of small molecules with a molecular weight lower than 200 amu.

Such an inaccuracy of the previous solvation model for large molecules can be understood in terms of the two points. First, the roles of intramolecular interactions in solvation were neglected in the solvation energy function although they could become important in large molecules because of the increase in the number of neighboring atoms in molecular structure. A significant enhancement in the accuracy is therefore expected by the introduction of a proper self-solvation term in the solvation energy function. Second, the increase in molecular size can make the chemical environments around a solute atom too complicated for it to be described properly with one of the existing 23 atom types. The accuracy of the present GA-based solvation model is likely to be enhanced in a straightforward way by subdividing the atom types in such a way that the extended atomic parameter space can discriminate all the solute atoms in different chemical environments. Therefore, we expect that the improvement of the solvation free energy function under consideration of the above two points will increase its accuracy to a large extent and provide a reliable method for estimating the solvation energies of organic molecules with biological/physical activities.

We now turn to the new solvation model involving the self-solvation term and the extended atom types. The potential energy function in this solvation model differs from that of the previous one in that the atom types for the majority of carbon, nitrogen, and oxygen atoms are subdivided according to the number of substituents at the central atoms. The atomic parameter space extended in this way seems to be capable of discriminating the atoms with different steric hindrances for the accessibility of solvent molecules. Table 2 lists the optimized atomic parameters for the extended 37 atom types. It appears to be a common feature for various atom types that the *V*_*i*_ value of a solute atom decreases with the increase in the number of substituents. This indicates that the highly substituted solute atoms should have a small solvent-exposed volume as a consequence of the increased steric hindrance by the neighboring groups.

**Table 2:** **The optimized atomic fragmental volume (*V_j_*), maximum atomic occupancy (*O_i_***^**max**^**), atomic solvation (*S_i_*), and atomic desolvation (*P_i_*) parameters in the solvation model including self-solvation effects**

**Atom type**	**Description**	***V***_***j***_**(Å**^**3**^**)**	**O**_***i***_^**max**^**(Å**^**3**^**)**	***S***_***i***_**(kcal/molÅ**^**3**^**)**	***P***_***i***_**(kcal/molÅ**^**3**^**)**
C.3_4	sp^3^ carbon with 4 substituents	6.342	316.5	0.310	−0.905
C.3_3	sp^3^ carbon with 3 substituents	7.977	321.4	0.302	−1.432
C.3_2	sp^3^ carbon with 2 substituents	9.365	334.2	0.270	−2.055
C.3_1	sp^3^ carbon with 1 substituent	11.341	343.0	1.937	−3.095
C.2_3	sp^2^ carbon with 3 substituents	8.571	322.3	−0.429	−1.397
C.2_2	sp^2^ carbon with 2 substituents	9.365	338.2	1.914	−2.476
C.2_1	sp^2^ carbon with 1 substituent	10.952	352.5	−1.381	−3.846
C.1_2	sp carbon with 2 substituents	8.968	325.3	−0.730	−3.201
C.1_1	sp carbon with 1 substituent	17.698	342.8	1.857	−3.286
C.ar_3	aromatic carbon with 3 substituents	7.342	346.9	1.324	−3.095
C.ar_2	aromatic carbon with 2 substituents	9.762	354.2	0.032	−3.787
N.3_3	sp^3^ nitrogen with 3 substituents	6.587	314.5	−9.680	20.746
N.3_2	sp^3^ nitrogen with 2 substituents	7.853	327.3	−15.984	19.305
N.3_1	sp^3^ nitrogen with 1 substituent	10.952	341.5	−16.776	10.095
N.2	sp^2^ nitrogen	8.275	328.7	−8.079	5.643
N.1	sp nitrogen	10.952	364.3	−8.540	9.968
N.am_3	amidic nitrogen with 3 substituents	6.164	348.2	−10.619	7.286
N.am_2	amidic nitrogen with 2 substituents	8.653	356.2	−12.571	16.220
N.am_1	amidic nitrogen with 1 substituent	11.349	368.3	−20.952	20.048
N.ar	aromatic nitrogen	8.243	338.3	−7.937	8.397
N.pl3_3	planar nitrogen with 3 substituents	6.984	321.8	−1.587	5.873
N.pl3_2	planar nitrogen with 2 substituents	8.245	330.6	−14.021	9.143
N.pl3_1	planar nitrogen with 1 substituent	10.556	338.4	−14.571	9.422
O.3_2	sp^3^ oxygen with 2 substituents	6.532	363.8	−7.831	9.571
O.3_1	sp^3^ oxygen with 1 substituent	5.397	371.6	−15.089	12.857
O.2	sp^2^ oxygen	7.833	344.3	−5.032	4.873
S.3	sp^3^ sulfur	16.896	340.6	−1.905	−2.307
S.2	sp^2^ sulfur	17.143	348.3	1.190	9.397
S.O	sulfoxide sulfur	13.810	345.2	−5.857	1.381
S.O2	sulfone sulfur	11.905	338.2	4.315	3.984
P	phosphorine	10.029	330.2	5.286	−17.857
F	fluorine	8.454	326.0	1.365	−2.303
Cl	chlorine	16.905	326.3	0.435	−2.937
Br	bromine	22.460	330.4	0.185	−5.073
H.C	hydrogen bonded to carbon	2.714	366.4	−0.476	−6.048
H.N	hydrogen bonded to nitrogen	1.786	364.8	4.730	−17.863
H.O	hydrogen bonded to oxygen	5.571	362.1	6.524	−22.032

The overall interactions between the solute carbon atoms and solvent molecules are predicted to be repulsive in the present solvation model because eight and three of their eleven atom types have positive and small negative *S*_*i*_ values, respectively, which is consistent with the immiscibility of hydrocarbons in water. On the other hand, the negative values for all their optimized *P*_*i*_ parameters imply that even the neutral carbon atoms can make a significant contribution to the stabilization of organic molecules in aqueous solution. This stabilization effect should apparently be attributed to the attractive intramolecular hydrophobic interactions between the nonpolar groups. Such a role of intramolecular hydrophobic interactions in the stabilization of a solute molecule in aqueous solution was also implicated in molecular dynamics simulation studies with generalized Born force field [39]. These intramolecular hydrophobic interactions were shown to become more important in self-solvation with the increase in molecular size of the solute [40]. Despite the abundance of the neutral carbon atoms in organic molecules, however, the low absolute values of their *P*_*i*_ parameters indicate that the intramolecular hydrophobic interactions should be insufficient by themselves to be the major driving force to stabilize the solute molecules in solution.

It is interesting to note that the unsubstitued and partially substituted polar atoms have even more negative *S*_*i*_ values than the fully substituted ones as can be seen in the case of N.3, N.am, N.pl3, and O. 3 in Table 2. This implies that the former can interact with solvent molecules in more attractive manner than the latter. The weakening of solute-solvent interactions for highly substituted polar atoms can be attributed to the increase in the excluded volume, which has an effect of preventing the solvent molecules from approaching the central solute atoms. It is also noteworthy that all of the nitrogen and oxygen atoms appear to have negative *S*_*i*_ and positive *P*_*i*_ values. This suggests that they can be stabilized in solution only by the intermolecular solute-solvent interactions rather than the intramolecular interactions between solute atoms. Most noticeably, the *S*_*i*_ parameters of the hydrogen atoms bonded to nitrogen and oxygen are found to be positive in the present solvation model whereas their corresponding *P*_*i*_ values are negative. These results indicate the preference for the formation of intramolecular hydrogen bonds between solute atoms over the intermolecular solute-solvent ones in the case that a group in the solute molecule should play the role of hydrogen bond donor. Such a difficulty for the solvent molecules to serve as a hydrogen bond acceptor with respect to the solute atoms may be attributed to the presence of better hydrogen bond acceptors or to the rarity of better hydrogen bond donors in the solute than water. This is consistent with the results of recent molecular dynamics simulation studies in which the solute-solvent hydrogen bonds were shown to be dynamically more stable when the water molecules played a role of a hydrogen bond donor than when they served as an acceptor [41]. Thus, the *P*_*i*_ values of hydrogen atoms also exemplify the importance of intramolecular interactions in stabilizing the organic molecules in solution and the self-solvation term in the solvation free energy function.

**Figure 4:**
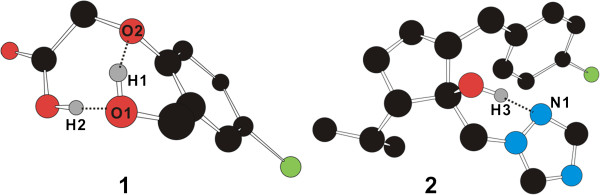
**The structures of 1 ((4-chloro-2-hydroxymethyl-phenoxy)-acetic acid) and 2 (2-(4-chloro-benzyl)-5-isopropyl-1-**[1,2,4]triazol-1-ylmethyl-cyclopentanol) optimized at B3LYP/6-31G* level of theory with PCM solvation model. Carbon, hydrogen, nitrogen, oxygen, and chloride atoms are indicated in black, gray, blue, red, and green, respectively. Each dotted line indicates a hydrogen bond. Hydrogen atoms attached to carbons are omitted for visual clarity.

The limited role of solvent molecules in the hydrogen-bond stabilization of solutes can be related with the fact that a strong hydrogen bond is more difficult to be established in solution than in the gas phase due to the role of rupturing or weakening the hydrogen bonds played by water molecules [42,43]. The extent of this negative solvent effect should be greater in the intermolecular solute-solvent hydrogen bond than in the intramolecular one because the former is exposed to bulk solvent in a larger part than the latter. In other words, the intramolecular hydrogens bonds have a better chance to be protected in solution than the solute-solvent ones due to the presence of the more neighboring solute atoms that can limit the approach of solvent molecules. Figure 4 illustrates the structures of two molecules included in the training set, **1** ((4-chloro-2-hydroxymethyl-phenoxy)-acetic acid) and **2** (2-(4-chloro-benzyl)-5-isopropyl-1-[1,2,4]triazol-1-ylmethyl-cyclopentanol), optimized at B3LYP/6-31G* level of theory with polarizable continuum model for solvation. Two strong intramolecular O···H···O hydrogen bonds appear to play a crucial role in stabilizing **1** in aqueous solution whereas **2** can be stabilized in solution by establishing one intramolecular O···H···N hydrogen bond and simultaneously the hydrophobic interactions between its nonpolar groups. These optimized structures exemplify the significant role of intramolecular hydrogen bonds in the stabilization of organic molecules in solution.

**Figure 5:**
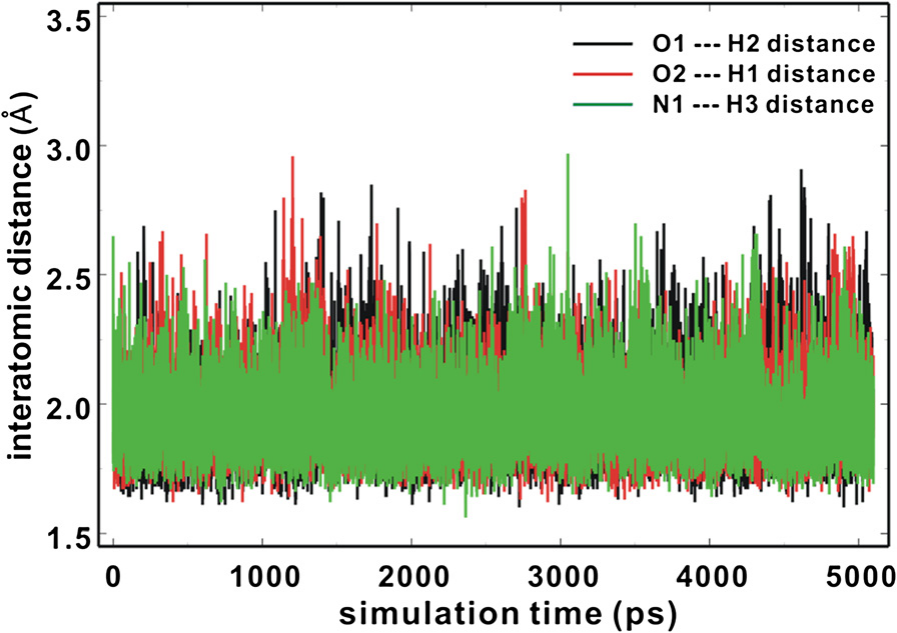
**Time evolutions of the interatomic distances associated with the intramolecular hydrogen bond interactions in 1 and 2. See Figure **4** for the identification of atoms.**

As a check for the stabilities of the intramolecular hydrogen bonds shown in Figure 4, we performed molecular dynamics simulations of **1** and **2** in aqueous solution with AMBER force field [44] and explicit solvent model (TIP3P) [45]. Figure 5 displays the time dependences of the interatomic distances associated with the three intramolecular hydrogen bonds. All three hydrogen bonds seem to be dynamically stable during the entire course of simulation time (5.1 ns) with the associated time-averaged hydrogen-bond distance of 1.92 – 1.96 Å. Indeed, the three hydrogen bonds are maintained for more than 95% of simulation time when the distance limit for O–H···O and O–H···N hydrogen bonds of moderate strength is assumed to be 2.2 Å [46]. These dynamic stabilities confirm the significant role of intramolecular hydrogen bonds in the stabilization of solutes by self-solvation.

**Figure 6:**
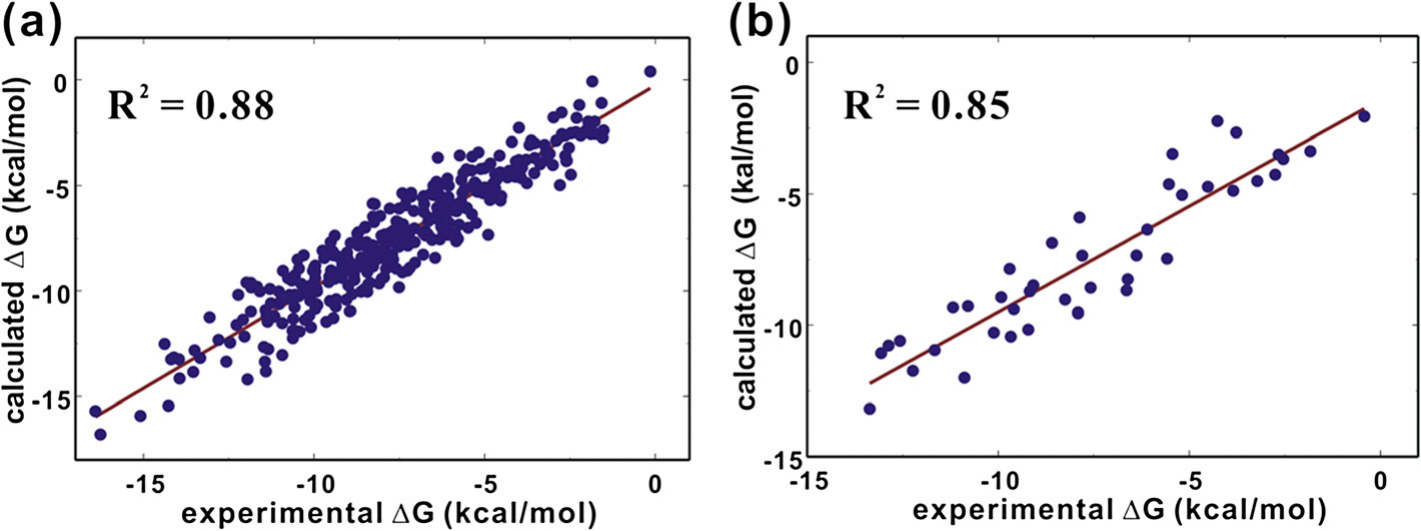
**Correlation diagrams for experimental solvation free energies (ΔG) versus those obtained with the solvation free energy function with self-solvation term for (a) 362 molecules in the training set and (b) 42 molecules in the test set.** The slope and intercept of the fitting for the test set are 1.05 and 0.31, respectively.

Figure 6 illustrates the correlations between the experimental solvation free energies and those calculated using Equation (6). Molecular structures, experimental and calculated solvation free energies of all 404 molecules are shown in Additional file 1. The number of molecules in the dataset may be insufficient to optimize total 148 atomic parameters. However, the reference dataset could not be extended further because of the limited number of molecules for which experimental solubility and vapor pressure data were available. We note that the *R*^*2*^ values for training and test sets increase substantially from 0.68 and 0.67 in the previous solvation model with 72 atomic parameters (Figure 3) to 0.88 and 0.85 in the present solvation model with 148 atomic parameters, respectively. As a check on the transferability of the optimized atomic parameters, we investigated the accuracy of the present solvation model with the dataset employed in the previous study [27]. The *R*^*2*^ values between the experimental and the calculated solvation free energies amount to 0.92 and 0.88 for the training set of 131 molecules and the test set of 24 molecules, respectively. These validation results indicate the transferability of the optimized parameters for organic molecules with varying sizes, shapes, and functional groups. Such a significant improvement in predictability may be attributed to the inclusion of the self-solvation term in the solvation model and to the extension of atom types to cope with various chemical environments in large molecules.

To address the relative importance of the self-solvation term and the extension of atom types in the accuracy of solvation free energy function, we compared the experimental solvation free energies of the molecules in the dataset to those obtained with the two solvation models involving only one of the two accuracy-enhancing factors. The *R*^*2*^ value for the test set in case of 37 atoms types without the self-solvation term and that in case of 23 atom types with the self-solvation term amount to 0.77 and 0.74, respectively. These similar *R*^*2*^ values indicate that the self-solvation effect and the extension of atomic parameters may contribute to the improvement in the accuracy of solvation free energy function to a similar extent.

The root mean square deviations of the solvation free energies estimated with Equation (6) from experimental results amount to 1.18 kcal/mol for the SAMPL1 test set. This result is better than those of SM6, SM8, and SMD continuum solvation models tested using the same dataset [26] although they could give better results when the different datasets were used in the validation [47,48]. The accuracy of the present solvation model is also comparable in terms of R^2^ value to those of the quantum chemical dielectric continuum solvation model (COSMO) [17] and molecular dynamics free energy perturbation method [11]. These comparisons indicate that our GA-based solvent-contact model may be more efficient in a quantitative estimation of molecular solvation free energies than the sophisticated quantum chemical method and statistical simulations with all-atom models because the former can produce the energy values in a straightforward way from the simplified potential function.

To further address the effects of including the self-solvation term and the extension of atom types on the accuracy of solvation free energy function, we calculated the mean absolute deviation (MAD), maximum absolute deviation (XAD), mean relative absolute deviation (MRAD), and maximum relative absolute deviation (XRAD) values between the experimental solvation free energies of the molecules in the test sets and those calculated with various solvation models. As shown in Table 3, all four deviation parameters decrease due either to the inclusion of the self-solvation term or to the extension of atomic parameters. When the two factors are considered together, MAD and MRAD values fall to 1.17 kcal/mol and 20.64%, respectively. These results confirm the necessity for both the self-solvation term and the extension of atom types of the solute atoms to enhance the accuracy of solvation free energy function.

**Table 3:** Comparisons of mean absolute deviation (MAD), maximum absolute deviation (XAD), mean relative absolute deviation (MRAD), and maximum relative absolute deviation (XRAD) between the experimental solvation free energies of the molecules in the test set and those calculated with various solvation models

**Solvation model**	**MAD**	**XAD**	**MRAD**	**XRAD**
	**(kcal/mol)**	**(kcal/mol)**	**(%)**	**(%)**
23 atom types without self-solvation term	1.72	4.88	32.40	303.25
37 atom types without self-solvation term	1.32	4.06	31.85	171.83
23 atom types with self-solvation term	1.43	3.80	28.19	221.76
37 atom types with self-solvation term	1.17	2.09	20.64	90.73

In comparison with the results for the previous solvation model that neglected the self-solvation effect, the largest improvements in solvation free energies are observed for the molecules in which the strong intramolecular hydrogen bonds or intramolecular van der Waals contacts can be established. For example, the differences between the experimental and calculated solvation free energies for **1** and **2** in Figure 4 decrease from 4.8 and 4.3 kcal/mol in the previous solvation model to 0.2 and 0.1 kcal/mol in the present method, respectively, due to the inclusion of the self-solvation term and to the extension of atom types. This substantial improvement further exemplifies the importance of intramolecular interactions in the stabilization of organic molecules in solution, which was also proposed for the structural stability of proteins in solution [42].

The earlier solvation models such as solvent accessible surface area, hydrophobicity scales, and group additivity models proved to be insufficient to explain the solvation properties of organic molecules [49]. This has been attributed to the assumption that molecular solvation free energy could be approximated as the sum of fragmental contributions to solvation. Such a group additivity criterion for solute-solvent interactions is actually inapplicable to large organic molecules because their buried regions can also contribute to the structural stability in solution. Our modified solvent-contact model confirmed that the solvation free energies of large organic molecules could be estimated with reasonable accuracy by combining the contributions from the solvent-exposed and self-solvation regions, the relative importance of which should be dependent on the molecular conformations in solution. The significant contribution of the self-solvation term to solvation free energies of organic molecules is thus consistent with the nonadditivity in solute-solvent interactions that stems from the intramolecular interactions between solute atoms in solution.

Despite the improved accuracy in estimating the solvation free energies of organic molecules, some problems still remain for the present solvation model to be employed extensively in practical applications. First, the atomic parameters of some atom types such as the cationic carbon and the hydrogen attached to sulfur atom could not be determined in this study due to the lack of corresponding experimental data. The atomic parameter space needs to be extended to cover a more variety of atom types in molecules when the more experimental data for molecular solvation free energies will be available in the future. Second, conformational diversity of organic molecules should be considered in the parameterization because the volumes of solvent-exposed and buried regions can vary with the conformational changes. For this purpose, molecular dynamics or Monte Carlo simulations can be applied prior to the parameterization to collect various local structural minima of the solutes. Finally, the solvation free energy function needs to be decomposed into enthalpy and entropy terms. Because both thermodynamic quantities are experimentally accessible, the potential parameters in the enthalpic and entropic terms can be optimized independently using their respective corresponding experimental data. Apparently, this dual parameterization warrants the better correlation between the experimental and computational solvation free energies than the single parameterization because more diverse experimental data can be included in reference dataset. Because the sign of solvation free energy is determined by the combination of enthalpic and entropic contributions, the decomposition analysis of solvation free energy can also provide thermodynamic insight into the solvation mechanism. Our future studies for solvation will focus on further improvement in the accuracy of solvation free energy function with the three above-mentioned points kept in mind.

## Conclusions

We have shown the superiority of our modified solvent-contact model to the previous one in predicting the molecular solvation free energies of various organic molecules. The improvement in the accuracy could be attributed to the inclusion of the self-solvation term in the solvation free energy function and to the extension of the atom types to cope with a variety of chemical environments. The newly constructed solvation free energy function included total 148 atomic parameters for 37 atom types. All these parameters could be optimized by the operation of a standard genetic algorithm using the experimental solvation free energy data for 362 organic molecules with varying sizes and shapes and their 3-D atomic coordinates obtained from quantum chemical geometry optimization at B3LYP/6-31G* level of theory. As a consequence of the modifications, the *R*^*2*^ values between the experimental and calculated solvation free energies increased from 0.68 and 0.67 to 0.88 and 0.85 for training and test sets, respectively. This significant enhancement in the accuracy confirmed the importance of intramolecular interactions and the inherence of nonadditivity in molecular solvation, which had also been implicated in the precedent experimental and theoretical studies on solute-solvent interactions. Considering the simplicity in energy calculation and model refinement, we expect that the present solvation model can be useful for the estimations of the solubility and desolvation cost for organic molecules in aqueous solution.

## Competing interests

The authors declare that they have no competing interests.

## Authors’ contributions

HC: Development of methodology, HK: C++ Programming to run genetic algorithm, HP: Developed idea and wrote paper. All authors read and approved the final manuscript.

## Supplementary Material

Additional file 1 Contains chemical structures, experimental and calculated solvation free energies of 404 molecules used in this study. Click here for file
