# Supplementary material for: New solvation free energy function comprising intermolecular solvation and intramolecular self-solvation terms
Source: J Cheminform. 2013 Feb 4;5:8. doi: 10.1186/1758-2946-5-8 (PMC3573996; doi:10.1186/1758-2946-5-8)
Supplement: Additional file 1 — Contains chemical structures, experimental and calculated solvation free energies of 404 molecules used in this study. [file 1758-2946-5-8-S1.doc]

**Experimental (ΔG^s^_exp_) and calculated (ΔG^s^_calc_) solvation free energies (in kcal/mol) of the 362 compounds in the training set.**

| chemical structure  molecule name | | ΔG^s^_exp_ | | | ΔG^s^_calc_ | | chemical structure  molecule name | | ΔG^s^_exp_ | | ΔG^s^_calc_ | | |  |
| --- | --- | --- | --- | --- | --- | --- | --- | --- | --- | --- | --- | --- | --- | --- |
|  | | -7.77 | | | -7.24 | |  | | -4.83 | | -3.95 | | |  |
|  | | -7.95 | | | -9.35 | |  | | -12.22 | | -10.18 | | |  |
|  | | -7.41 | | | -9.11 | |  | | -10.33 | | -9.74 | | |  |
|  | | -5.29 | | | -4.84 | |  | | -10.56 | | -10.17 | | |  |
|  | | -6.54 | | | -7.57 | |  | | -6.65 | | -6.67 | | |  |
|  | | -7.65 | | | -7.59 | |  | | -7.92 | | -7.30 | | |  |
|  | | -5.62 | | | -4.40 | |  | | -12.8 | | -12.32 | | |  |
|  | | -7.84 | | | -7.58 | |  | | -4.99 | | -5.08 | | |  |
|  | | -2.04 | | | -2.42 | |  | | -7.69 | | -8.47 | | |  |
| chemical structure  molecule name | | ΔG^s^_exp_ | | | ΔG^s^_calc_ | | chemical structure  molecule name | | ΔG^s^_exp_ | | ΔG^s^_calc_ | | |  |
|  | | -3.09 | | | -3.48 | |  | | -7.83 | | -8.61 | | |  |
|  | | -7.3 | | | -7.36 | |  | | -5.38 | | -5.06 | | |  |
|  | | -6.04 | | | -6.76 | |  | | -10.37 | | -9.66 | | |  |
|  | | -8.82 | | | -9.47 | |  | | -8.03 | | -8.32 | | |  |
|  | | -4.87 | | | -5.48 | |  | | -13.34 | | -13.18 | | |  |
|  | | -8.41 | | | -8.52 | |  | | -7.33 | | -5.93 | | |  |
|  | | -6.78 | | | -6.24 | |  | | -10.26 | | -11.37 | | |  |
|  | | -9.27 | | | -9.30 | |  | | -10.56 | | -11.88 | | |  |
|  | | -10.57 | | | -10.76 | |  | | -6.41 | | -6.44 | | |  |
| chemical structure  molecule name | | ΔG^s^_exp_ | | | ΔG^s^_calc_ | | chemical structure  molecule name | | ΔG^s^_exp_ | | ΔG^s^_calc_ | | |  |
|  | | -8.26 | | | -8.71 | |  | | -6.81 | | -5.57 | | |  |
|  | | -7.98 | | | -7.58 | |  | | -7.08 | | -6.19 | | |  |
|  | | -3.95 | | | -4.28 | |  | | -7.35 | | -7.53 | | |  |
|  | | -4.2 | | | -2.93 | |  | | -7.42 | | -7.52 | | |  |
|  | | -8.65 | | | -8.84 | |  | | -4.89 | | -7.33 | | |  |
|  | | -10.61 | | | -11.32 | |  | | -7.68 | | -8.50 | | |  |
|  | | -11.67 | | | -9.80 | |  | | -3.24 | | -3.07 | | |  |
|  | | -3.37 | | | -3.98 | |  | | -7.22 | | -7.38 | | |  |
|  | | -9.43 | | | -8.30 | |  | | -9.41 | | -10.60 | | |  |
| chemical structure  molecule name | | ΔG^s^_exp_ | | | ΔG^s^_calc_ | | chemical structure  molecule name | | ΔG^s^_exp_ | | ΔG^s^_calc_ | | |  |
|  | | -6.46 | | | -5.45 | |  | | -5.65 | | -5.34 | | |  |
|  | | -7.19 | | | -8.89 | |  | | -7.4 | | -7.74 | | |  |
|  | | -2.79 | | | -4.97 | |  | | -10.14 | | -11.17 | | |  |
|  | | -11.59 | | | -10.01 | |  | | -7.99 | | -8.44 | | |  |
|  | | -9.51 | | | -7.68 | |  | | -5.82 | | -5.89 | | |  |
|  | | -4 | | | -3.86 | |  | | -10.76 | | -9.39 | | |  |
|  | | -5 | | | -4.17 | |  | | -3.53 | | -2.99 | | |  |
|  | | -2.18 | | | -2.62 | |  | | -5.2 | | -5.28 | | |  |
|  | | -7.51 | | | -6.76 | |  | | -7.59 | | -8.93 | | |  |
| chemical structure | | ΔG^s^_exp_ | | | ΔG^s^_calc_ | | chemical structure | | ΔG^s^_exp_ | | ΔG^s^_calc_ | | |  |
|  | | -3.78 | | | -3.76 | |  | | -7.89 | | -8.94 | | |  |
|  | | -7.25 | | | -7.22 | |  | | -9.57 | | -9.57 | | |  |
|  | | -4.84 | | | -5.44 | |  | | -14.11 | | -13.15 | | |  |
|  | | -6.74 | | | -7.14 | |  | | -11.47 | | -12.66 | | |  |
|  | | -2.63 | | | -3.58 | |  | | -5.52 | | -6.05 | | |  |
|  | | -2.6 | | | -3.83 | |  | | -7.46 | | -7.50 | | |  |
|  | | -5.85 | | | -7.30 | |  | | -8.36 | | -9.61 | | |  |
|  | | -15.09 | | | -15.91 | |  | | -2.74 | | -1.54 | | |  |
|  | | -14.27 | | | -15.44 | |  | | -6.05 | | -4.99 | | |  |
| chemical structure | | ΔG^s^_exp_ | | | ΔG^s^_calc_ | | chemical structure | | ΔG^s^_exp_ | | ΔG^s^_calc_ | | |  |
|  | | -9.45 | | | -8.12 | |  | | -6.23 | | -5.55 | | |  |
|  | | -2.73 | | | -2.56 | |  | | -13.06 | | -11.25 | | |  |
|  | | -9.45 | | | -9.27 | |  | | -7.5 | | -7.22 | | |  |
|  | | -9.92 | | | -9.89 | |  | | -9.11 | | -8.36 | | |  |
|  | | -8.33 | | | -8.46 | |  | | -8.13 | | -8.31 | | |  |
|  | | -9.39 | | | -9.41 | |  | | -5.91 | | -6.17 | | |  |
|  | | -9.01 | | | -8.47 | |  | | -6.31 | | -7.54 | | |  |
|  | | -9.05 | | | -10.41 | |  | | -11.16 | | -11.20 | | |  |
|  | | -6.21 | | | -7.14 | |  | | -6.39 | | -6.04 | | |  |
| chemical structure | | ΔG^s^_exp_ | | | ΔG^s^_calc_ | | chemical structure | | ΔG^s^_exp_ | | ΔG^s^_calc_ | | |  |
|  | | -7.91 | | | -7.90 | |  | | -11.41 | | -13.82 | | |  |
|  | | -11.42 | | | -10.91 | |  | | -10.91 | | -9.04 | | |  |
|  | | -4.1 | | | -4.57 | |  | | -6.03 | | -5.88 | | |  |
|  | | -7.67 | | | -7.29 | |  | | -7.59 | | -7.75 | | |  |
|  | | -4.73 | | | -5.15 | |  | | -9.96 | | -9.40 | | |  |
|  | | -10.42 | | | -11.92 | |  | | -6.27 | | -6.63 | | |  |
|  | | -10.48 | | | -8.50 | |  | | -8.51 | | -7.85 | | |  |
|  | | -6.25 | | | -6.73 | |  | | -4.66 | | -4.60 | | |  |
|  | | -6.5 | | | -4.99 | |  | | -10.71 | | -10.18 | | |  |
| chemical structure | | ΔG^s^_exp_ | | | ΔG^s^_calc_ | | chemical structure | | ΔG^s^_exp_ | | ΔG^s^_calc_ | | |  |
|  | | -5.78 | | | -7.52 | |  | | -8.95 | | -10.96 | | |  |
|  | | -4.69 | | | -5.11 | |  | | -6.37 | | -5.18 | | |  |
|  | | -8.6 | | | -8.63 | |  | | -6.77 | | -5.87 | | |  |
|  | | -7.66 | | | -7.22 | |  | | -5.79 | | -5.99 | | |  |
|  | | -3.93 | | | -4.63 | |  | | -6.06 | | -5.54 | | |  |
|  | | -8.73 | | | -7.27 | |  | | -6.09 | | -4.66 | | |  |
|  | | -8.18 | | | -8.51 | |  | | -9.99 | | -8.87 | | |  |
|  | | -9.03 | | | -8.17 | |  | | -0.14 | | 0.40 | | |  |
|  | | -6.56 | | | -7.50 | |  | | -2.32 | | -2.47 | | |  |
| chemical structure | | ΔG^s^_exp_ | | | ΔG^s^_calc_ | | chemical structure | | ΔG^s^_exp_ | | ΔG^s^_calc_ | | |  |
|  | | -7.58 | | | -6.69 | |  | | -2.46 | | -2.51 | | |  |
|  | | -4.08 | | | -3.84 | |  | | -4.53 | | -5.70 | | |  |
|  | | -7.06 | | | -7.02 | |  | | -6.14 | | -4.84 | | |  |
|  | | -9.18 | | | -9.37 | |  | | -1.51 | | -2.39 | | |  |
|  | | -6.38 | | | -4.88 | |  | | -1.88 | | -2.55 | | |  |
|  | | -2.59 | | | -3.81 | |  | | -5.04 | | -5.35 | | |  |
|  | | -8.28 | | | -5.85 | |  | | -9.18 | | -8.92 | | |  |
|  | | -10.18 | | | -10.15 | |  | | -3.19 | | -3.77 | | |  |
|  | | -6.27 | | | -6.94 | |  | | -8.7 | | -7.84 | | |  |
| chemical structure | | ΔG^s^_exp_ | | | ΔG^s^_calc_ | | chemical structure | | ΔG^s^_exp_ | | ΔG^s^_calc_ | | |  |
|  | | -5.72 | | | -6.02 | |  | | -3.63 | | -2.87 | | |  |
|  | | -5.69 | | | -5.75 | |  | | -8.02 | | -7.37 | | |  |
|  | | -3.54 | | | -4.40 | |  | | -4.33 | | -4.60 | | |  |
|  | | -11.34 | | | -12.76 | |  | | -13.94 | | -14.14 | | |  |
|  | | -6.64 | | | -6.52 | |  | | -8.44 | | -9.10 | | |  |
|  | | -6.14 | | | -6.38 | |  | | -13.96 | | -13.24 | | |  |
|  | | -1.54 | | | -2.72 | |  | | -2.9 | | -2.87 | | |  |
|  | | -4.54 | | | -4.36 | |  | | -8.5 | | -6.99 | | |  |
|  | | -3.9 | | | -4.13 | |  | | -6.06 | | -7.14 | | |  |
| chemical structure | | ΔG^s^_exp_ | | ΔG^s^_calc_ | | | chemical structure | | ΔG^s^_exp_ | | | ΔG^s^_calc_ | |  |
|  | | -6.37 | | -6.27 | | |  | | -9.96 | | | -10.84 | |  |
|  | | -10.08 | | -10.16 | | |  | | -6.73 | | | -7.36 | |  |
|  | | -8.24 | | -7.99 | | |  | | -5.38 | | | -4.27 | |  |
|  | | -10.1 | | -9.73 | | |  | | -9.75 | | | -8.08 | |  |
|  | | -10.56 | | -9.25 | | |  | | -3.99 | | | -3.78 | |  |
|  | | -8.47 | | -7.74 | | |  | | -8.46 | | | -7.68 | |  |
|  | | -11.26 | | -10.58 | | |  | | -8.83 | | | -9.24 | |  |
|  | | -8.24 | | -6.72 | | |  | | -9.9 | | | -10.67 | |  |
|  | | -6.06 | | -5.65 | | |  | | -9.39 | | | -7.37 | |  |
|  | chemical structure | | ΔG^s^_exp_ | | | ΔG^s^_calc_ | | chemical structure | | ΔG^s^_exp_ | | | ΔG^s^_calc_ | |
|  |  | | -4.74 | | | -4.88 | |  | | -5.11 | | | -4.20 | |
|  |  | | -9.96 | | | -9.07 | |  | | -9.96 | | | -8.79 | |
|  |  | | -8.61 | | | -8.41 | |  | | -12.57 | | | -13.35 | |
|  |  | | -7.29 | | | -5.59 | |  | | -10.11 | | | -10.45 | |
|  |  | | -9.7 | | | -8.43 | |  | | -6.57 | | | -5.37 | |
|  |  | | -7.3 | | | -7.15 | |  | | -5.98 | | | -5.98 | |
|  |  | | -6.66 | | | -7.30 | |  | | -1.57 | | | -1.09 | |
|  |  | | -6.8 | | | -7.23 | |  | | -6.45 | | | -8.42 | |
|  |  | | -7.26 | | | -9.03 | |  | | -9.55 | | | -9.32 | |
|  | chemical structure | | ΔG^s^_exp_ | | | ΔG^s^_calc_ | | chemical structure | | ΔG^s^_exp_ | | | ΔG^s^_calc_ | |
|  |  | | -8.27 | | | -8.83 | |  | | -8.53 | | | -9.92 | |
|  |  | | -10.62 | | | -12.24 | |  | | -12.27 | | | -11.61 | |
|  |  | | -11.84 | | | 9.83 | |  | | -8.39 | | | -8.23 | |
|  |  | | -8.75 | | | -8.74 | |  | | -4.51 | | | -5.49 | |
|  |  | | -4.84 | | | -5.67 | |  | | -2.22 | | | -1.19 | |
|  |  | | -11.85 | | | -10.97 | |  | | -3.2 | | | -3.42 | |
|  |  | | -14.19 | | | -13.24 | |  | | -8.27 | | | -6.46 | |
|  |  | | -3.76 | | | -4.87 | |  | | -7.15 | | | -5.51 | |
|  |  | | -8.09 | | | -9.64 | |  | | -4.69 | | | -4.44 | |
|  | chemical structure | | ΔG^s^_exp_ | | | ΔG^s^_calc_ | | chemical structure | | ΔG^s^_exp_ | | | ΔG^s^_calc_ | |
|  |  | | -12.04 | | | -12.16 | |  | | -7.41 | | | -5.35 | |
|  |  | | -6.8 | | | -8.27 | |  | | -8.01 | | | -9.41 | |
|  |  | | -9.17 | | | -8.72 | |  | | -6.29 | | | -6.29 | |
|  |  | | -9.37 | | | -9.21 | |  | | -9.28 | | | -9.09 | |
|  |  | | -5.6 | | | -5.21 | |  | | -11.95 | | | -14.19 | |
|  |  | | -7.39 | | | -6.83 | |  | | -3.99 | | | -2.26 | |
|  |  | | -7.04 | | | -8.65 | |  | | -16.41 | | | -15.69 | |
|  |  | | -14.38 | | | -12.51 | |  | | -9.36 | | | -10.73 | |
|  |  | | -9.21 | | | -9.04 | |  | | -3.68 | | | -3.07 | |
|  | chemical structure | | ΔG^s^_exp_ | | | ΔG^s^_calc_ | | chemical structure | | ΔG^s^_exp_ | | | ΔG^s^_calc_ | |
|  |  | | -7.38 | | | -6.96 | |  | | -5.53 | | | -4.22 | |
|  |  | | -6.32 | | | -5.54 | |  | | -9.54 | | | -8.01 | |
|  |  | | -12.46 | | | -12.45 | |  | | -6.41 | | | -4.81 | |
|  |  | | -5.37 | | | -6.35 | |  | | -9.32 | | | -8.34 | |
|  |  | | -2.64 | | | -2.62 | |  | | -2.98 | | | -1.77 | |
|  |  | | -5.24 | | | -4.49 | |  | | -8.03 | | | -8.16 | |
|  |  | | -8.06 | | | -6.43 | |  | | -9.91 | | | -10.01 | |
|  |  | | -5.68 | | | -4.73 | |  | | -9.93 | | | -10.62 | |
|  |  | | -5.25 | | | -6.77 | |  | | -16.26 | | | -16.79 | |
|  | chemical structure | | ΔG^s^_exp_ | | | ΔG^s^_calc_ | | chemical structure | | ΔG^s^_exp_ | | | ΔG^s^_calc_ | |
|  |  | | -7.67 | | | -7.13 | |  | | -5.53 | | | -6.07 | |
|  |  | | -8.97 | | | -8.97 | |  | | -2.3 | | | -1.80 | |
|  |  | | -10.65 | | | -9.50 | |  | | -1.95 | | | -2.20 | |
|  |  | | -8.62 | | | -10.03 | |  | | -8.7 | | | -7.21 | |
|  |  | | -6.25 | | | -6.01 | |  | | -2.53 | | | -3.20 | |
|  |  | | -1.96 | | | -1.97 | |  | | -5.48 | | | -6.99 | |
|  |  | | -11.44 | | | -13.35 | |  | | -1.84 | | | -0.07 | |
|  |  | | -1.77 | | | -1.97 | |  | | -8.28 | | | -7.82 | |
|  |  | | -7.78 | | | -6.08 | |  | | -8.62 | | | -8.65 | |
|  | chemical structure | | ΔG^s^_exp_ | | | ΔG^s^_calc_ | | chemical structure | | ΔG^s^_exp_ | | | ΔG^s^_calc_ | |
|  |  | | -8.9 | | | -8.71 | |  | | -8.27 | | | -7.92 | |
|  |  | | -6.52 | | | -5.43 | |  | | -11.36 | | | -11.46 | |
|  |  | | -8.92 | | | -7.72 | |  | | -7.45 | | | -6.96 | |
|  |  | | -7.51 | | | -7.89 | |  | | -10.45 | | | -10.24 | |
|  |  | | -11.03 | | | -10.02 | |  | | -7.25 | | | -7.84 | |
|  |  | | -13.5 | | | -12.82 | |  | | -12.08 | | | -11.36 | |
|  |  | | -9.35 | | | -10.39 | |  | | -8.89 | | | -10.15 | |
|  |  | | -9.53 | | | -10.47 | |  | | -5.22 | | | -5.27 | |
|  |  | | -9.43 | | | -11.29 | |  | | -10.3 | | | -8.30 | |
|  | chemical structure | | ΔG^s^_exp_ | | | ΔG^s^_calc_ | | chemical structure | | ΔG^s^_exp_ | | | ΔG^s^_calc_ | |
|  |  | | -10.65 | | | -10.42 | |  | | -8.93 | | | -10.95 | |
|  |  | | -10.44 | | | -8.93 | |  | | -8.5 | | | -6.95 | |
|  |  | | -7.15 | | | -5.34 | |  | | -10.52 | | | -10.22 | |
|  |  | | -4.65 | | | -4.71 | |  | | -10.98 | | | -10.49 | |
|  |  | | -8.68 | | | -5.87 | |  | | -6.35 | | | -6.00 | |
|  |  | | -8.22 | | | -7.62 | |  | | -7.46 | | | -7.05 | |
|  |  | | -2.47 | | | -4.47 | |  | | -6.37 | | | -3.67 | |
|  |  | | -3 | | | -4.02 | |  | | -11.16 | | | -11.22 | |
|  |  | | -7.69 | | | -7.15 | |  | | -10.03 | | | -9.04 | |
|  | chemical structure | | ΔG^s^_exp_ | | | ΔG^s^_calc_ | | chemical structure | | ΔG^s^_exp_ | | | ΔG^s^_calc_ | |
|  |  | | -6.43 | | | -6.55 | |  | | -11.85 | | | -9.61 | |
|  |  | | -5.73 | | | -3.56 | |  | | -5.27 | | | -5.13 | |
|  |  | | -5.18 | | | -3.42 | |  | | -9.64 | | | -11.47 | |
|  |  | | -9.47 | | | -9.12 | |  | | -11.39 | | | -11.16 | |
|  |  | | -6.07 | | | -7.33 | |  | | -11.29 | | | -9.90 | |
|  |  | | -3.53 | | | -3.68 | |  | | -4.56 | | | -3.57 | |
|  |  | | -5.36 | | | -3.62 | |  | | -8.99 | | | -9.73 | |
|  |  | | -13.54 | | | -13.84 | |  | | -10.09 | | | -11.72 | |
|  |  | | -10.93 | | | -11.53 | |  | | -7.71 | | | -8.26 | |
|  |  | | -10.63 | | | -11.88 | |  | | -4.28 | | | -4.29 | |
|  | chemical structure | | ΔG^s^_exp_ | | | ΔG^s^_calc_ | | chemical structure | | ΔG^s^_exp_ | | | ΔG^s^_calc_ | |
|  |  | | -7.51 | | | -9.82 | |  | | -6.78 | | | -5.49 | |
|  |  | | -10.93 | | | -13.04 | |  | | -1.75 | | | -2.54 | |
|  |  | | -9.5 | | | -9.02 | |  | | -9.15 | | | -8.87 | |
|  |  | | -5.82 | | | -6.21 | |  | | -7.05 | | | -7.68 | |
|  |  | | -8.52 | | | -8.50 | |  | | -8.44 | | | -8.65 | |
|  |  | | -5.75 | | | -6.85 | |  | | -8.44 | | | -10.03 | |
|  |  | | -6.81 | | | -7.21 | |  | | -7.51 | | | -6.08 | |
|  |  | | -8.41 | | | -9.68 | |  | | -11.98 | | | -9.60 | |
|  |  | | -11.02 | | | -10.61 | |  | | -5.83 | | | -7.63 | |

**Experimental (ΔG^s^_exp_) and calculated (ΔG^s^_calc_) solvation free energies (in kcal/mol) of the 42 compounds in the test set.**

| chemical structure | ΔG^s^_exp_ | ΔG^s^_calc_ | chemical structure | ΔG^s^_exp_ | ΔG^s^_calc_ |
| --- | --- | --- | --- | --- | --- |
|  | -10.79 | -9.27 |  | -10.88 | -12.00 |
|  | -11.18 | -9.32 |  | -5.53 | -4.62 |
|  | -6.64 | -8.67 |  | -2.66 | -3.50 |
|  | -2.75 | -4.26 |  | -7.91 | -9.55 |
|  | -13.36 | -13.18 |  | -3.77 | -2.66 |
|  | -9.59 | -9.39 |  | -6.1 | -6.35 |
|  | -9.92 | -8.93 |  | -5.58 | -7.46 |
|  | -7.91 | -9.51 |  | -9.7 | -7.85 |
|  | -12.57 | -10.60 |  | -6.38 | -7.34 |
|  | -9.67 | -10.44 |  | -2.54 | -3.67 |
|  | -13.06 | -11.07 |  | -9.17 | -8.71 |
|  | -5.19 | -5.03 |  | -12.23 | -11.74 |
| chemical structure | ΔG^s^_exp_ | ΔG^s^_calc_ | chemical structure | ΔG^s^_exp_ | ΔG^s^_calc_ |
|  | -7.58 | -8.57 |  | -11.66 | -10.95 |
|  | -7.87 | -5.89 |  | -10.11 | -10.29 |
|  | -8.59 | -6.86 |  | -12.87 | -10.78 |
|  | -4.26 | -2.23 |  | -7.8 | -7.35 |
|  | -8.25 | -9.02 |  | -3.22 | -4.50 |
|  | -0.42 | -2.05 |  | -9.08 | -8.48 |
|  | -9.21 | -10.17 |  |  |  |
|  | -1.83 | -3.37 |  |  |  |
|  | -6.61 | -8.25 |  |  |  |
|  | -3.85 | -4.87 |  |  |  |
|  | -4.51 | -4.72 |  |  |  |
|  | -5.44 | -3.47 |  |  |  |
